# Consent revisited: the impact of return of results on participants' views and expectations about trial participation

**DOI:** 10.1111/hex.12371

**Published:** 2015-04-30

**Authors:** Carolyn Tarrant, Clare Jackson, Mary Dixon‐Woods, Sarah McNicol, Sara Kenyon, Natalie Armstrong

**Affiliations:** ^1^ SAPPHIRE Department of Health Sciences University of Leicester Leicester UK; ^2^ The Education & Social Research Institute Manchester Metropolitan University Manchester UK; ^3^ School of Health and Population Sciences College of Medical and Dental Sciences University of Birmingham Edgbaston Birmingham UK

**Keywords:** clinical trial, decision‐making, informed consent, qualitative, research results, UK

## Abstract

**Background:**

Increasingly, the sharing of study results with participants is advocated as an element of good research practice. Yet little is known about how receiving the results of trials may impact on participants' perceptions of their original decision to consent.

**Objective:**

We explored participants' views of their decision to consent to a clinical trial after they received results showing adverse outcomes in some arms of the trial.

**Method:**

Semi‐structured interviews were conducted with a purposive sample of 38 women in the UK who participated in a trial of antibiotics in pregnancy. All had received results from a follow‐up study that reported increased risk of adverse outcomes for children of participants in some of the trial intervention arms. Data analysis was based on the constant comparative method.

**Results:**

Participants' original decisions to consent to the trial had been based on hope of personal benefit and assumptions of safety. On receiving the results, most made sense of their experience in ways that enabled them to remain content with their decision to take part. But for some, the results provoked recognition that their original expectations might have been mistaken or that they had not understood the implications of their decision to participate. These participants experienced guilt, a sense of betrayal by the maternity staff and researchers involved in the trial, and damage to trust.

**Conclusions:**

Sharing of study results is not a wholly benign practice, and requires careful development of suitable approaches for further evaluation before widespread adoption.

## Background

Informed consent is the cornerstone of ethical research practice; providing potential participants with full information is seen as critical in promoting autonomous decision making and preventing exploitation.^1^ There is extensive evidence, however, that participants' decisions to take part in research are often not ‘fully informed’, but instead are based on trust, altruism and expectations of personal benefit,^2^, ^3^, ^4^, ^5^ and may involve misunderstandings about study design.^6^, ^7^ Although the possibility of decisions to participate being based on false understandings has received extensive attention,^8^, ^9^ as, separately, have participants' preferences and expectations for receiving results of studies in which they have participated^10^, ^11^, ^12^, ^13^ and the emotional impact of receiving those,^14^, ^15^ little attention has been given to how people's views of the consent they gave on joining a study may be affected by their learning of the results. This is an important void given that providing the results of research to participants is increasingly framed as an ethical obligation for researchers.^16^, ^17^ In this article, we present empirical evidence of how receiving the results of a clinical trial can cause participants to revisit their decision to take part.

### Why do people take part in medical research?

Decisions to participate in medical research are characteristically founded on the notion of a co‐operative bargain, underpinned by assumptions that the values and interests of those involved in conducting the research are aligned with participants' own.^5^ These assumptions include expectations that research will contribute to medical knowledge and improved treatment for future patients, and hence serve the public good, and that there will be systems for regulating and monitoring research to ensure safety – which indeed there are. Participants also rely heavily on trust; they trust that research will be conducted in a proper and ethical manner, but they also draw on trust and confidence in the wider fields of medicine and health care, within which they expect to be protected and cared for.^5^

This raises the question of what happens when people receive study findings that appear to conflict with the expectations that underpinned their decision to participate. This is a particular concern in randomized controlled trials (RCTs) where the study design means there is always a risk that outcomes will not be as good as participants might have hoped.

#### Randomized controlled trials

RCTs enable systematic investigation of whether a given treatment confers a benefit for patients compared with controls – either another treatment(s) or a placebo. Although trials are only conducted when there are reasonable grounds to expect benefit from the trialled interventions, and no evidence of harm, new treatments are found to be better than existing ones just over half the time, and new treatments may indeed perform less well.^18^ Any group that does less well in a situation specifically aimed at determining which is the best intervention may feel disadvantaged – regardless of whether they are in the control or intervention group, and in all trials there is the possibility that some participants will experience unanticipated adverse outcomes or harm (Box 1). It is only when participants receive the results of the trial that they can compare their outcomes to ‘what could have beenߣ, and the assumptions and expectations underpinning their decision to take part may come under scrutiny.

Box 1 **Hypothetical trial and outcomes**

**Simplified hypothetical trial testing new drug A for rheumatoid arthritis**

Participants are randomized into either an intervention group, which receives drug A, or a control group which receives usual care (drug B). The primary outcome is disability associated with disease progression at one year following treatment initiation. Possible trial outcomes include

1. Levels of disability in patients in the intervention group (drug A) are significantly lower than those in the control group (drug B) thereby indicating that trial drug A offers benefit to patients.
2. Levels of disability in patients in the intervention group (drug A) are not significantly different to those in the control group (drug B), thereby indicating that trial drug A offers no benefits over usual care.
3. Levels of disability in patients in the intervention group (drug A) are not significantly different to those in the control group (drug B), but a higher risk of suffering severe migraines is found in the intervention group. This indicates that trial drug A offers no benefits over usual care, but is associated with an increased risk of migraine.
4. Levels of disability in patients in the intervention group (drug A) are significantly higher than those in the control group (drug B), thereby indicating that trial drug A is less effective than usual care.

### The Oracle Children's Study

We use an example of a study that found an increased risk of adverse outcomes for participants in the intervention arms of a trial. The study was a long‐term follow‐up of children born to participants in the ORACLE trial, an RCT that investigated the use of two broad‐spectrum antibiotics for women with suspected pre‐term labour.^19^, ^20^ The trial was designed to generate new evidence about the optimal management of women at risk of pre‐term labour. The treatment being tested in the trial – antibiotics – was a promising intervention for which there was some prior, but not definitive, evidence of benefit. The RCT found some benefits and some risks of antibiotics in the short term. However, the subsequent long‐term follow‐up study – the Oracle Children's Study – unexpectedly found antibiotics to be associated with increased risks of functioning problems and a small increased risk of cerebral palsy in children whose mothers had had suspected pre‐term labour with intact membranes (SPL), as well as a small increased risk of bowel problems in children whose mothers had pre‐term rupture of the membranes (PROM)^21^, ^22^ (see Boxes 2 and 3). Extensive work was carried out to ensure that the return of results was handled in an appropriate and ethical way, and procedures were put in place to support women on receipt of the results (including a national telephone helpline).^23^

It is the Oracle Children's follow‐up study that is our focus of interest. Based on interviews with a purposeful sample of women who elected to receive the results of this study, we explore how women revisited their decision to take part in the original ORACLE trial in the light of receiving the findings and describe the emotional sequelae of this

Box 2 **The ORACLE trial**

The ORACLE trial evaluated the effects of prescribing erythromycin or co‐amoxiclav for women with either preterm rupture of the membranes (‘waters breaking’ prior to 37 weeks pregnancy) (PROM) or spontaneous preterm labour with intact membranes (contractions starting prior to 37 weeks of pregnancy) (SPL), and with no overt infection, using a 2 × 2 factorial design.

**Findings**

For women with PROM

1. Erythromycin was associated with prolongation of pregnancy and improvements in short‐term maternal and neonatal morbidity; for singletons, there was a reduction in the composite primary outcome (death or abnormal cerebral ultrasound or use of supplemental oxygen at 36 weeks post‐menstrual age).
2. Co‐amoxiclav was associated with increased risk of neonatal necrotizing enterocolitis.

For women with SPL

1. There was no evidence of either benefit or harm at discharge from hospital.

Box 3 **The follow‐up ORACLE Children Study**

The ORACLE Children Study sought follow‐up information for surviving children at 7 years of age in the UK using a parent‐report postal questionnaire. Primary outcome was defined as the presence of any level of functional impairment using the Multi‐Attribute Health Status (MAHS) classification system. Secondary outcomes included a range of medical and behavioural outcomes.

Educational attainment at 7 years was assessed for children resident in England using results from National Curriculum tests at Key Stage 1.

**Findings**

For children whose mothers had PROM

1. There was little evidence of long‐term effects of prescription of antibiotics on the health and educational attainment of children at 7 years.

For children whose mothers had SPL

1. Prescription of erythromycin was associated with an increase in the proportions of children with any level of functional impairment from 38 to 42%.
2. There was an increase in the proportions of children with cerebral palsy from 1.7 to 3.3% associated with erythromycin and from 1.9 to 3.2% with co‐amoxiclav.
3. There was a suggestion that more children who developed cerebral palsy had been born to mothers who had received both antibiotics in combination.

## Methods

We conducted semi‐structured interviews with 38 women who had participated in the ORACLE Children's Study, received the results of that study in the form of a feedback leaflet (see supplementary material 1) and responded to an invitation to interview that accompanied the leaflet. Women living within 100 miles of Leicester (UK) were purposively sampled to ensure diversity in terms of emotional reactions to the leaflet (self‐reported in a postal questionnaire) and whether their child was affected by any of the conditions mentioned in the leaflet (a functional impairment, cerebral palsy and/or a bowel problem).

Semi‐structured interviews were carried out by CJ, a social scientist trained in qualitative methods. An interview prompt guide was developed based on a review of the literature and project team discussions, and was used flexibly in interviews. Interviews focused on participants' reactions to receiving the study findings, and their feelings about having participated in the ORACLE trial. Women were not informed about the trial arm to which they had been allocated, and nor was the interviewer or any other member of the project team. Interviews were conducted in participants' homes. Each interview lasted approximately 1 h and was digitally recorded.

Analysis was based on the constant comparative method.^24^ Initial open coding by SM informed the development of a thematic coding framework grounded in the data. The framework was reviewed by the research team and was used to code all data systematically in NVivo. Data summaries were produced for high‐level themes. Quotations in the text are labelled to indicate whether the participant had PROM or SPL, and whether their child was affected (A) or not affected (NA) by any of the conditions mentioned in the Oracle Children's Study feedback leaflet.

The interview study was given ethics approval by the West Midlands NHS Research Ethics Committee.

## Findings

### Participants

Thirty‐eight women were interviewed, aged between 28 and 59 years (average age 39). All but three were of ‘white British’ or ‘white Irish’ ethnicity. Two‐thirds (25) had been recruited into the ORACLE trial due to pre‐term rupture of the membranes (PROM), 13 due to spontaneous pre‐term labour (SPL).

At the time of interview, the children born to participants in the ORACLE trial ranged from 8 to 13 years old (average 10 years). Six participants in the interviews reported their ORACLE child had no health problems; the children of the remaining 32 had a range of health problems including cerebral palsy (5), other neurological problems (6), learning difficulties (8), bowel problems (3), respiratory problems (6), psychological or emotional difficulties (9), physical functioning problems (5) and visual problems (5).

### The decision to participate

Women's accounts of their decisions to consent to the ORACLE trial of antibiotics demonstrated that they drew heavily on a hope of personal benefit and assumptions of safety. While many women were able to reconcile the findings of the follow‐up study with their original decision to participate, for others the return of results was disruptive and led them to question the basis of their decision to consent to the trial.

### Decision making about participation

Women reported that they had been recruited to the ORACLE trial on arriving in hospital with premature labour or broken waters. They were potentially anxious, stressed and in pain. Despite staff receiving careful training to talk to women and stress the importance of informed consent, and women being provided with an information sheet about the trial approved by the ethics committee, most of the women in our study described having made the decision to take part without much consideration. Their motivation for participating was primarily a hope that it might help their baby; women tended to orient to the trial as an individual intervention which had the possibility of bringing improved outcomes. This individualistic perspective was often coupled with altruistic motives.

Anything's a bonus if it's gonna help then we've got to try it. […] I was hoping and praying that it would help (Interviewee 16, PROM, NA)

For me it was important that I participate in the trial in order to help other women not have to go through what I went through (Interviewee 37, SPL, A)

The accounts of women showed that they had also relied on a heuristic^25^ about safety – a mental ‘shortcut’ to facilitate decision making – that had enabled them to consent to the trial without having to engage in complex reasoning at a time when they were feeling anxious and vulnerable. Women expected, at best, that taking part in the trial would potentially help their baby, and at worst, it might make no difference: this expectation was strongly reinforced by their perceptions of antibiotics as a routine and familiar medication. Although the official leaflets approved for the trial explained that there could be risks associated with taking part, women described relying on ‘common sense’ understandings of what they were signing up to, including an implicit assumption that there was little or no risk involved.^26^ Only three of the 38 women reported having considered that there might be negative outcomes: these women described having explicitly weighed up the risks of taking part in a trial with the potential for benefit to their baby. The remainder did not recall considering the possibility of any risks.

I just remember saying yes straight away. I never thought about it, so, at the time I never thought that I was taking a risk. (Interviewee 3, PROM, A)

Women drew on expectations that the maternity staff caring for them would have their best interests at heart in inviting them to take part in the trial. Inherent in the accounts of many women was the assumption that any risk of harm would be known about in advance.

You just trust the people that are referring you through, because no way would they put you through on a trial knowing that you are pregnant with a life and it's gonna be detrimental to you and your child. (Interviewee 38, SPL, A)

Further, they drew on wider expectations of value congruence. They correctly perceived that those involved in medical research would be working for the good of patients, would avoid exposing patients to undue harm and would only subject them to interventions for which there was some expectation of benefit and where risk was expected to be low (as was the case for the antibiotics in the trial).

I assumed that they fully expected it to help and it would I suppose, otherwise I wouldn't have said yes. […] You put your trust in the medical profession and scientists all the time don't you? You have to assume that they're not gonna deliberately harm you or your child (Interviewee 11, PROM, NA)

For some, their perceptions of the cumulative nature of clinical research, and the strict controls governing research, provided further reassurance.

There's so much regulation and government guidelines and so on that you're half way there to knowing the answers. […] The chances are it's not gonna be really harmful (Interviewee 11, PROM, NA)

Women's accounts demonstrated that all these inferences came together to form a reasonable set of assumptions about safety.

### Heuristics about decision to participate preserved

Despite the negative and potentially distressing findings of the Oracle Children's Study, the majority of women still valued receiving the results. It enabled a sense of closure and completeness, and signified to women that the researchers were acknowledging their contribution.

It was like you'd not been forgotten about really, that all of us that had done this, we'd done it and it had been acknowledged by getting the results. (Interviewee 28, PROM, A)

For the majority of women (around three‐quarters of interviewees), receiving the results of the follow‐up study did not lead them to question the assumptions and expectations that they had relied upon in deciding to take part in the trial; they were able to affirm their original decision to participate. They acknowledged in hindsight that research involves uncertainty, and that the negative outcomes were unlucky and unforeseen. Some argued that such negative outcomes had to be balanced against the importance of the knowledge and medical advances generated through research.

I think it's important to do these things ‘cos it's like any trial, if people aren't willing to participate then it's very difficult to move forward. As hard as that may seem if some people have had a negative outcome, it's better to push things forward than always be afraid almost to do anything (Interviewee 12, PROM, NA)

Importantly, although the finding of negative outcomes was not what they hoped for, receiving the results affirmed that the research had been of value and would benefit other women in a similar position in the future. This meant for many women that one of the core foundations of their decision to take part – a desire to contribute to the ‘common good’ – remained intact. This enabled women to continue to feel good about having taken part; they experienced the warm glow of having helped others.

Just the thought that something that you've done might help somebody in your position in the future's a really good feeling. (Interviewee 10, PROM, A)

Some women made sense of the results by re‐interpreting them selectively, in ways that preserved their individual narrative about their decision to take part in the trial and subsequent outcomes for their child. In the feedback leaflet, women were informed that it was not possible to know whether trial participation had led to an individual child's health problems (and that many children who are born prematurely are already at risk from these health problems); for some women, this information about uncertainty was helpful. It allowed them to protect their beliefs that their child had benefitted from the research, or to suspend the possibility that the research might have harmed their child. As a result, the expectations that underpinned their decision to take part in the trial were preserved, and they were able to continue feeling that they had done the right thing.

It's just that little bit of doubt in my mind ‐ did [the trial] contribute towards [child's health problems …]? I just keep an open mind on that. […] If it does benefit people in future when their waters go or anything like I think it's a good thing really, so I've no regrets or anything (Interviewee 13, PROM/SPL, A)

Other women disregarded this information about uncertainty, and assumed a direct causal link between trial participation and their own child's health outcomes. For some women, this had positive consequences: although initially upsetting, it enabled them to make sense of their child's health or behaviour, and justify it to others.

It was pretty tough to get this result, but then good in a way, that now I know what's wrong with him […] and it kind of took a bit of the pressure off [child] (Interviewee 9, SPL, A)

For others, results were seeing as having little relevance; these women did not revisit their decision to take part in the trial, perhaps because they perceived that they were not in the ‘at risk’ group, their child did not have health problems, or they did not fully understand the results.

Did I remember rightly that it reduced [cerebral palsy] or that it doesn't affect? […] I think obviously I was in the other group which is probably why I didn't pay [attention] (Interviewee 05, PROM, A)

### Heuristics about decision to participate come under scrutiny

Although most women came to peace with their decision to participate in the trial following receipt of the results, around a quarter of the women reacted more negatively. These women experienced a sense of shock at the outcomes of the research. They were distressed by the discovery that by taking part in the trial they had exposed their child to a possible risk of harm, given that their decision to participate was based on the reasonable assumption that this would be a safe thing to do. For these women, receiving the results of the Oracle Children's Study led them to call into question the basis for their decision. This was associated with extremely negative emotions, including guilt, anger and a sense of betrayal by the maternity staff and researchers involved in the trial.

Many of these women's children had some form of health problem. Although their child's problems could have been caused by multiple factors, these women found it easy to attribute negative outcomes to trial participation. Counterfactual thinking – thinking about what might have been – was a common response.^27^ This led some women to experience significant feelings of regret and guilt about their decision to participate in the trial, as they now perceived that it might have been implicated in harm:

When we saw the potential that he could well have had the brain damage as a result of the trial that obviously brings up huge guilt emotion (Interviewee 08, SPL, A)

These reactions were not limited to women whose children had been affected by conditions listed in the leaflet giving the results of the follow‐up study. Some women whose children were not affected by a health problem empathized strongly with others, and felt that, although they had been lucky, it could have been their child.

The shock bit about the cerebral [palsy…], I was still part of that and that could have happened to [child] […] Of course you feel saddened but you still selfishly think ‘but I was okay and this worked out all right’. (Interviewee 02, SPL, NA)

The women who blamed themselves for putting their child at risk now regarded the assumptions they had made, and the heuristics they had used in making the decision to take part in the trial as unsound. Receiving the results of the study exposed to these women that the bargain they had entered into was not what they thought it was. They were regretful or angry that they had relied on assumptions about benefit and safety, and that they had not fully understood what they were signing up to.

I felt bad that I'd even done the trial, I thought by doing what I'd done […] I could have put my child at risk. (Interviewee 24, SPL, NA)

Others, however, still regarded their assumptions as valid. They interpreted the negative results as a sign that the doctors, nurses or researchers involved in the trial had failed to live up to what research participants could legitimately expect from them, rather than recognizing that negative results are an unexpected but inevitable possibility of any trial. Although those involved in coordinating the trial went to great lengths to ensure that recruitment was conducted in an ethical manner, and the trial did not breach ethical or regulatory standards in any way, these women experienced a profound breach of trust. They questioned the motives and actions of those involved in the research, feeling that they had been let down, misled or exploited when they were in a distressed and vulnerable position, by the very nursing and medical staff they trusted to care for them. They interpreted the negative outcomes as indicating that the doctors, nurses or researchers had not fulfilled their side of the co‐operative bargain and experienced this as a sense of betrayal.

I really felt as if I'd been cheated really, and fooled into taking something that I wouldn't have had I have known all the facts […] Angry for myself for […] trusting that everything was fine. (Interviewee 31, PROM, NA)

That was my primary concern that, hang on a minute, I was duped into this by a nurse in a maternity ward somewhere. (Interviewee 09 SPL, A)

They also questioned the regulatory systems that they had assumed would prevent this sort of thing from happening by ensuring drugs were safe before they were tested on patients.

Just shocked that they could give a drug like that and not fully know what the reaction would be to the unborn baby. […] You just wouldn't think something they're giving you would have repercussions, no you think that everything's been tested and everything's been trialled. (Interviewee 09, SPL, A)

Central to the accounts of these women was a belief that the risks involved could have been known ‘in advance’ and that only the conspiratorial, wilfully negligent or malevolent intentions of researchers could have been responsible for withholding them from gullible patients. Eight interviewees said they would definitely not take part in a trial again; their experiences had fundamentally undermined their trust in research, and they regretted the risk they felt that they had unwittingly taken.

I wouldn't do it, and there's no way I'd be used as a guinea pig again […] me and my child. (Interviewee 31, PROM, NA)

## Discussion

Trial outcomes may be concordant with participants' expectations and welcome, but they also have the potential to be dissonant and disruptive. Our analysis of sharing the results of a long‐term follow‐up of a trial of an intervention in pregnancy found that when study findings are not as expected or hoped for, participants have to find ways to make sense of these outcomes and reconcile them with the assumptions that underpinned their decision to take part. In the process, the majority affirmed their initial assumptions, but some came to question their decision and the behaviour and ethics of those involved in the trial.

Participants in the ORACLE trial accounted for their decision to participate by describing expectations of potential for individual benefit, altruistic motives and a reliance on heuristics of safety. Women drew on their trust in health professionals and medicine, and invoked a vaguely conceptualized regulatory structure, to underpin their beliefs that they would be protected from harm. The feeling of safety was enhanced by the familiarity and low threat associated with the study intervention (antibiotics). When it was unexpectedly found that antibiotics were associated with risk of harm in some children, participants had to work to reconcile this unwelcome discovery with their assumptions and beliefs.

Most were able to rationalize the results in ways that were consistent with a pre‐existing narrative about their participation and the subsequent outcomes for their child. For these women, their assumptions about the co‐operative bargain they had entered into in agreeing to take part in the research were not disrupted, and they were able to continue to feel positive about their experience of taking part. Yet some participants found it far harder to reconcile themselves to the findings. They responded by internalizing a sense of blame and experienced regret arising from a belief that more vigilance on their part when they consented would have avoided exposure to risk. Others experienced a sense of betrayal and undermining of their trust. They concluded that a lack of vigilance, a lack of concern for their well‐being, or even exploitation or malfeasance, on the part of researchers had resulted in their being exposed to risk; crucially, they did not recognize the nature of uncertainty involved in trials.

In consenting to research, participants describe drawing heavily on trust ‘borrowed’ from the status of health professionals and expectations of health care as offering the best available treatment when needed. However, the nature of clinical trials means that there is uncertainty in advance about what that treatment will be. If an intervention performs better than the control, then those in the control group may perceive themselves disadvantaged; the long‐term evidence suggests it is almost equally likely the same effect may occur for those exposed to unsuccessful trial interventions. Put in the vernacular, those in the least well‐performing arms of a trial may see themselves as the ‘losers’. When results are shared with participants, this discovery of the possibility of disadvantage can result in participants experiencing guilt and regret or feelings of betrayal, and can do damage to trust.^8^ Although the ORACLE trial had been designed to meet and exceed ethical and regulatory standards, some participants who received the results came to believe that misconduct must have occurred and that the research and professional community had failed to uphold participants' trust, rather than recognizing the negative results as an inevitable potential consequence of trial design. Damage to trust is particularly problematic for a number of reasons, not least the fact that public trust is critical to the social licence that enables medical research to happen.^28^, ^29^

Although regulations and guidance currently place a heavy emphasis on the need for participants to be informed about risk and burdens when they are recruited to in a trial, in practice participant information focuses on possible side‐effects of interventions, physical discomfort, inconvenience and anxiety during the trial itself.^28^, ^30^ Our study suggests that it is equally important to ensure participants understand the nature of the bargain they are entering into, the uncertainties and the risks of negative outcomes. Novel approaches to the process of informed consent, such as the use of decision aids, may be one way of achieving this.^31^, ^32^, ^33^ The need for improved training for healthcare staff to manage the tensions between their roles as clinicians and as recruiters to research has also been emphasized.^34^ Improvements to the consent process alone, however, are unlikely to be the full solution, as ideals of informed consent endorsed by ethical and regulatory bodies^35^, ^36^ may be unachievable in practice.^30^, ^37^ It is important that participants are able to make decisions that are not based on mistaken beliefs,^37^ and likely to be crucial to improving their ability to do so is greater public understanding of the nature of the co‐operative bargain involved in research participation, both in terms of the science of trials, and the norms and values that researchers are expected to adhere to. Despite initiatives to educate and raise public awareness about research,^38^, ^39^, ^40^, ^41^ and increased patient involvement in the design and conduct of trials,^42^ this is likely to remain challenging^43^ and should be a focus of future effort.

One key implication of our study is the need for researchers to recognize that there will always be the potential for the return of results to cause distress or doubt. Understanding the basis for reactions such as feelings of guilt and betrayal may help researchers prepare their feedback in ways that avoid undermining the assumptions that underpin decisions to take part in research: when trial results are not as hoped for, any feedback to participants should carefully re‐explain nature of clinical trials, and emphasize that the outcome(s) could not have been known about nor anticipated in advance. Further, researchers should anticipate the need to provide support for participants in the period following receipt of the results and to consider how damage to trust can be minimized and repaired. Unblinding participants as to trial arm allocation needs careful consideration, as this in itself can have positive and negative consequences.^44^ Involving participants in the design of feedback processes is likely to be helpful in anticipating and planning for reactions to results.^23^

### Strengths and limitations

We interviewed a sample of participants purposively chosen to represent women who experienced a range of emotional reactions to the results; this enabled us to characterize the different ways that participants make sense of their experience. Participants may not have been representative of all the women who participated in the ORACLE trial, and women whose children had health and functional problems were probably been over‐represented. It may not be possible to generalize our findings to trials where outcomes are of a different nature (e.g. benefit in the intervention arms of a trial). The women in our study had participated in a trial during pregnancy, and results related to their children, as such, the results may have been particularly emotive. Trials with more or less at stake, and with different types of intervention (e.g. new drugs as opposed to familiar medication), may generate different types of response. However, the underlying dynamics of the co‐operative bargain are likely to be universal.^5^

The nature of the ORACLE trial meant that women were recruited to the study when potentially distressed. This might mean they were particularly likely to be critical of those involved in the research. Women were recruited to the original trial around 10 years ago. This could have implications for their recall of their consent decision and introduce potential for recall bias. Results were sent to women by post; other means of feeding back results (e.g. face‐to‐face) may generate different reactions, and possibly be protective of the social relationships involved.^11^

## Conclusions

The potential for unexpected negative outcomes is inherent in any trial, and receiving the results can lead participants to revisit their consent decision. We found that many participants remained content with their decision despite unanticipated negative outcomes, but others experienced feelings of regret, guilt and a sense of betrayal. While efforts to ensure that participants are aware of the nature of the bargain they are entering into when they consent to a trial are critical, we may have to accept that this is not always possible, and that distress and narrative disruption may be unavoidable costs of providing feedback to participants. Sharing of study results appears on the face of it to be an ethical obligation, but it is not a wholly benign practice and requires careful development of suitable approaches for further evaluation before widespread adoption.

## Funding

The ORACLE Children Study was funded by the UK Medical Research Council. This work was supported by Wellcome Trust Investigator Award WT097899. Mary Dixon‐Woods' contribution to this paper was also supported by University of Leicester study leave at the Dartmouth Institute for Health Policy and Clinical Practice. Clare Jackson held a DRF Doctoral Research fellowship, supported by the National Institute for Health Research, from 2010 to 2013. The views expressed in this publication are those of the authors and not necessarily those of the NHS, the National Institute for Health Research or the Department of Health.

## Conflict of interest

SK was lead investigator of the ORACLE trial and the ORACLE Children Study. No conflict of interests has been declared by the other authors.

## Supporting information


**Data S1**. The ORACLE Children Study: Feedback Leaflet.
