# Supplementary material for: Consent revisited: the impact of return of results on participants' views and expectations about trial participation
Source: Health Expect. 2015 Apr 30;18(6):2042–53. doi: 10.1111/hex.12371 (PMC4737222; doi:10.1111/hex.12371)
Supplement: Supplementary file 1 — Data S1. The ORACLE Children Study: Feedback Leaflet. [file HEX-18-2042-s001.pdf]

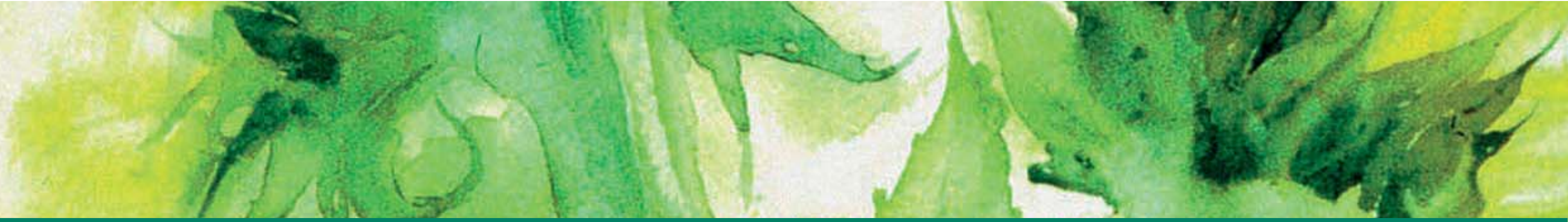

The ORACLE Children Study

A summary of the main results

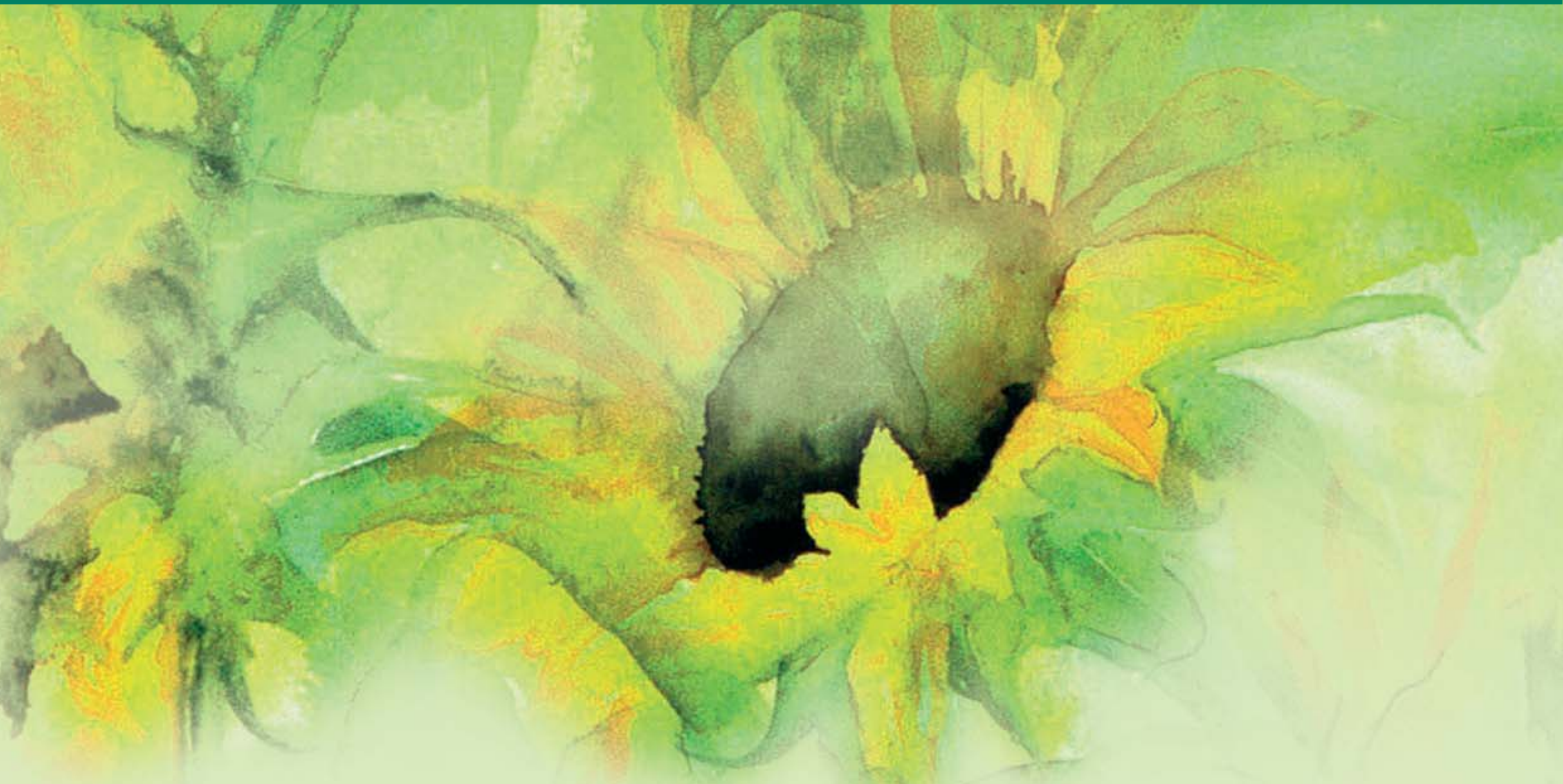

This leaflet contains a summary of the main results of the ORACLE Children Study. This leaflet has been written for parents who took part in the Study and presents the findings that are most likely to be interesting or important to parents. The full scientific papers are available from the ORACLE Children Study or by writing to the ORACLE team (details on reverse).

The ORACLE Children Study was a follow-up to the ORACLE Clinical Trial, which was conducted when women were pregnant. The Children Study followed children born to mothers who took part in the trial, looking at children's health and how they were doing at school when they were about 7 years old.

Prematurity in babies

Every year, about one in eight babies, or 80,000 a year, are born prematurely (before 37 weeks) in the UK. Prematurity is the most common reason for babies to die in the first month after birth. Premature babies who survive are more likely to have short-term problems such as breathing and feeding difficulties and infections. They may also have longer-term problems with functioning, behaviour and learning. Whatever happens, having a premature baby is often a traumatic and upsetting experience for women and their families.

Research, like the ORACLE Trial and Children Study, to try to find the best ways to treat premature labour and reduce short and long term problems is very important.

THE **ORACLE** CLINICAL TRIAL

What the ORACLE Clinical Trial aimed to do

Women who show signs of going into labour early or have early broken waters are at risk of premature birth. The ORACLE Clinical Trial aimed to find out whether it would be helpful for women in either of these situations to have antibiotics, as previous research had suggested mild infection in the membranes surrounding the baby might be involved. It was thought that antibiotics could help by either prolonging the pregnancy or improving the health of the baby, or both. But nobody knew for certain, and nobody knew which antibiotic might be the best. Doing a clinical trial is the best way of finding the answer to questions like these.

How the ORACLE Clinical Trial was designed

The ORACLE trial was interested in two antibiotics: erythromycin and co-amoxiclav (also known as "Augmentin"). It wanted to compare the effects of having erythromycin with the effects of not having erythromycin. It also wanted to compare the effects of having co-amoxiclav with not having it (see **Groups in the ORACLE Clinical Trial** below). Neither of these antibiotics was a "new" or experimental drug. They were already commonly being used to treat pregnant women and babies with infection before the trial started, but their effects were not fully understood in situations where women's waters had broken early or where women were showing signs of going into labour early without broken waters.

Women who were at risk of having a premature birth in either situation (early broken waters or signs of going into labour early) were asked to take part in the ORACLE trial. It was explained to women that:

> *If you are in premature labour, where your waters have not broken, the effect of antibiotics on either prolonging your pregnancy or the health of your baby is unknown. If your waters have broken early antibiotics are likely to prolong your pregnancy and reduce infection in you and your baby, but it is not known if this will make any difference to your baby's chances of survival and healthy development. This study will help to answer these questions. (From the leaflet about the ORACLE trial given to women when they were asked to take part.)*

Women who joined the trial had ONE of the following: co-amoxiclav on its own; or erythromycin on its own; or both antibiotics (erythromycin and co-amoxiclav); or placebo (sugar pills with no antibiotics in them). The treatment each woman had was chosen at random by a computer, so the effects of the different treatments could be compared fairly. "Placebos" were used to compare the effects of antibiotics with having no active treatment. All the tablets (whether they were antibiotics or not) looked identical and everyone was asked to take two tablets at a time. Women took the tablets for 10 days or until they gave birth (whichever was sooner).

Groups in the ORACLE Clinical Trial

To analyse the results, researchers grouped women into those who had erythromycin and those who had co-amoxiclav.

The Erythromycin Group: Women who had any erythromycin as part of the trial were in the "Erythromycin Group". Women could be in this Group in one of two ways: they had erythromycin on its own, or they had it with co-amoxiclav.

The Co-amoxiclav Group: Women who had any co-amoxiclav as part of the trial were in the "Co-amoxiclav group". Women could be in this Group in one of two ways: they had co-amoxiclav on its own, or they had it with erythromycin.

Researchers also looked at the effects of having two antibiotics together.

Results of the original ORACLE Trial: summary

The trial ran in 161 hospitals across the world between 1994 and 2000. In the end, 11,050 women took part in the ORACLE trial: 6,241 had possible early labour and 4,809 had early broken waters. The ORACLE trial made some significant discoveries. Importantly, the results of the trial were different for the women whose waters had broken early and the women who were showing signs of going into labour without broken waters.

Results of the original ORACLE Trial: women whose waters broke early

The antibiotic erythromycin seemed to be helpful for babies in the short-term. In the Erythromycin Group, babies were more likely to stay in the womb longer, and were less likely to need help with breathing, to have infections, or to have an abnormal brain scan.

The other antibiotic, co-amoxiclav, also increased the time babies stayed in the womb and reduced their need for oxygen. But the trial discovered an important problem with co-amoxiclav. There was a small increase in the number of babies born with a rare, sometimes very serious bowel condition called necrotising enterocolitis (NEC) to women with broken waters who were in the Co-amoxiclav Group. Because of its large size and design, the ORACLE trial was able to identify this rare risk, even though co-amoxiclav had previously been routinely used in pregnancy without reported problems.

Results of the original ORACLE Trial: women who had signs of going into labour early but didn't have broken waters

The antibiotics used in the trial (erythromycin and co-amoxiclav) did not have any short-term effects (good or bad) for babies born to women with signs of early labour without broken waters.

Impact of the ORACLE Trial

The ORACLE trial means that a lot more is now known about the best way to treat women who may be going into labour early. Without the thousands of women who participated in the ORACLE trial, these important discoveries would never have been made. As a result of the original ORACLE trial, many maternity units now give erythromycin to women with early broken waters. Antibiotics are NOT recommended for women who are possibly going into early labour, but do not have broken waters. Use of co-amoxiclav in pregnancy is discouraged.

THE **ORACLE** CHILDREN STUDY

The original ORACLE trial finished in 2000. The next stage was the ORACLE Children Study.

The ORACLE Children Study

The ORACLE Children Study began in 2002 and aimed to see how ORACLE children were doing around the age of 7. The original ORACLE trial only looked at the health of the woman and baby immediately after birth. It was always planned to follow-up the ORACLE children to see if there were any long-term effects of having or not having antibiotics. The Children Study only collected follow-up information. No treatments were given to mothers or their children as part of the Children Study.

The ORACLE Children Study asked mothers who had taken part in the original ORACLE trial to fill in a postal questionnaire about their child's health and how they were doing at school when their ORACLE child was around 7 years old.

What was measured in the ORACLE Children Study

The questionnaire aimed to find out about children's health, wellbeing and educational attainment. These are areas where there may sometimes be problems in any baby born early.

Functioning: The questionnaire asked about the child's vision, hearing, speech, walking, dexterity (using their hands), emotions, pain, and aspects of thinking such as learning and remembering. Children were classed by researchers as having a 'functioning' difficulty if parents reported that children had any difficulties at all in any of these areas. As a result, 'functioning' difficulties can range from the child having minor difficulties (things like wearing glasses, requiring a hearing aid, not always being completely happy, or having some difficulty when trying to solve day to day problems), to more severe problems (such as being unable to walk, see, hear or speak, or having severe difficulties with thinking and remembering).

Health problems and behaviour: The questionnaire asked about chest symptoms, hospital admissions, fits/seizures, and specific medical conditions (including cerebral palsy), as well as behaviour, emotions, hyperactivity, and relationships with others.

Educational attainment: The National Curriculum (Key Stage 1) tests for reading, writing and maths, which are undertaken by children aged 7 in England, were used to assess how well the children were doing at school.

The results of the ORACLE Children Study are based only on the information that parents provided in their questionnaires and on the Key Stage 1 results. No information was collected from doctors or other health professionals.

Results of the ORACLE Children Study

In the ORACLE Clinical Trial in the UK, 4148 children were born to mothers with early broken waters, and the Children Study was able to follow up 3171 (75%) of these children. In the UK, 4221 children were born to women with signs of early labour without broken waters, and the Children Study was able to follow up 3196 (71%) of these children.

The results of the ORACLE Children Study were different depending on whether women had early broken waters or were showing signs of going into labour early when they took part in the ORACLE trial.

Results of the ORACLE Children Study: women whose waters broke early

- For mothers whose waters broke early, having antibiotics (erythromycin or co-amoxiclav) in the ORACLE trial did not make any difference to their child's functioning, behaviour, most medical conditions, or Key Stage 1 results.
- Parents were specifically asked about bowel problems because of the findings of the ORACLE trial (mentioned earlier). The Children Study found that in the Co-amoxiclav Group, there was a small increase in the number of children with bowel problems (2.7%) compared with those who were not given co-amoxiclav at all (1.6%). However, the bowel problems in children reported by these parents were mostly not serious (such as constipation).

There is, therefore, little evidence of long term effects (good or bad) for children of women who had antibiotics when their waters broke early, but there is a need for caution about use of co-amoxiclav in this situation.

Results of the ORACLE Children Study: women who had signs of going into labour early without broken waters.

- For mothers who had signs of early labour without broken waters, having antibiotics (erythromycin or co-amoxiclav) did not make any difference to their child's behaviour, most medical conditions, or Key Stage 1 results.
- Functioning problems are common in children whose mothers showed signs of early labour without broken waters. It is normal for about 40% (4 in every 10) children born in this situation to have some kind of functioning problem. Being in the Co-amoxiclav Group did not make any difference to children's functioning. However, in the Erythromycin Group, there were slightly more children with a 'functioning' problem compared with those whose mothers who did not have any erythromycin. These functioning problems included very mild difficulties (see "What was measured in the ORACLE Children Study" box), and most of the functioning problems reported by parents were minor.

Functioning problems in children

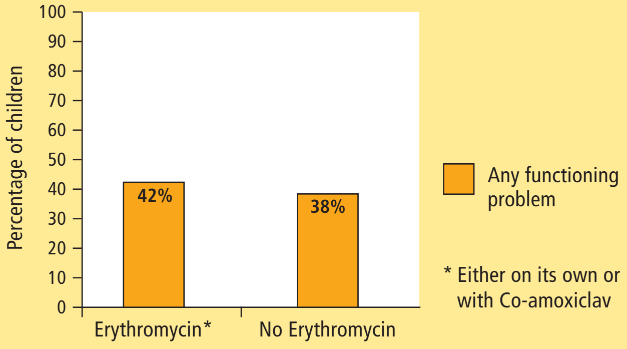

The ORACLE Children Study also made a very important and unexpected discovery about cerebral palsy that was only found in women who had early labour without broken waters. Before the ORACLE Children Study, it was known that cerebral palsy is more common in children whose mothers were at risk of premature labour, although it is a rare condition. In the group of mothers in the ORACLE trial who did not have an antibiotic at all (just placebos), 1.6% of children had cerebral palsy.

- It was found that cerebral palsy in children was somewhat more common when women with signs of early labour did have antibiotics. In the Erythromycin Group, 3.3% of children had cerebral palsy compared with 1.7% of children whose mothers who did not have erythromycin. In the Co-amoxiclav Group, 3.2% of children had cerebral palsy compared with 1.9% of children whose mothers who did not have any co-amoxiclav.

Cerebral palsy in children

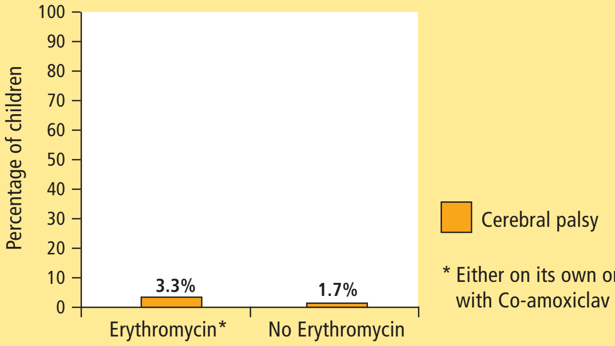

* Either on its own or with Co-amoxiclav

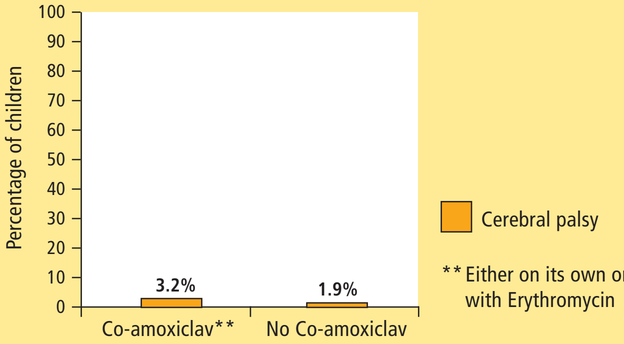

** Either on its own or with Erythromycin

- Looking at it in more detail, the increase in the number of children with cerebral palsy was clearest in the group of women who had both antibiotics (co-amoxiclav and erythromycin together). In this group, 4.4% of children had cerebral palsy, though even here the numbers with cerebral palsy remain quite small.

It is important to say that, even in the "both antibiotics" group, it was still very rare for children to have cerebral palsy. Cerebral palsy can have a number of different causes, including being born prematurely. We will never know what specifically caused the cerebral palsy in any individual child whose mother took part in the trial, only that there was a small increased risk (but not a definite cause) for women who had both antibiotics.

What is Cerebral Palsy?

Cerebral palsy is not a disease or an illness. It is the description of a physical impairment disorder that affects movement. The movement problems vary from mild to extremely severe. Cerebral palsy is most commonly the result of failure of a part of the brain to develop, either before birth or in early childhood. This is sometimes because of a blocked blood vessel, complications in labour, extreme prematurity or illness just after birth. Infections during pregnancy, or infancy and early childhood, e.g. meningitis or encephalitis, can also cause cerebral palsy. It is usually diagnosed early in life and does not occur for the first time when the child is older.

Many people with cerebral palsy are hardly affected. Others have problems walking, feeding, talking or using their hands. Some people are unable to sit up without support and need constant help. Sometimes other parts of the brain are also affected, causing sight problems, varying degrees of deafness and learning difficulties.

Adapted from the SCOPE website www.scope.org.uk

Impact of the ORACLE Children Study

The findings of the original ORACLE trial and the Children Study that followed are of great importance. Healthcare professionals, together with women, will be able to make the decisions that are right for women and their babies when they are at risk of having a premature baby.

The findings of the ORACLE trial and Children Study suggest that erythromycin may have some short-term, but not long-term, benefits for children whose mothers have early broken waters. The findings also suggest that antibiotics should not be given to women who are showing signs of going into labour early but do not have broken waters and do not have an obvious infection.

These findings do not mean that antibiotics are unsafe for use in pregnancy. Pregnant women with an infection should take antibiotics if they are given them. Where there is obvious infection, antibiotics may be life-saving for both mother and baby.

None of the findings could be known in advance. Without the help of women taking part in the ORACLE trial and the ORACLE Children Study, these discoveries about the effects of women taking antibiotics when they have signs of going into early labour or with early broken waters would not be known. These findings will make a difference to future generations.

Thank you very much for your participation, which has led to such important findings.

Some people may find the results of the ORACLE Children Study upsetting or may have questions. If you want to discuss these results further, contact your GP (who has been sent a letter about these findings) or the Helpline on 0800 085 2411. The Helpline number will be available in the short term between 9.30am and 4.30pm. If someone is not available to answer your call, please do leave a message with your telephone number and your call will be returned as soon as possible.

QUESTIONS AND ANSWERS

What is a clinical trial?

A clinical trial aims to make a fair comparison of different treatments. For comparisons to be fair, the groups of participants given different treatments should be similar so that the only difference between the groups is the different treatments. The best way of achieving this is to assign participants randomly to different treatment groups. It is also important to "blind" both doctors and participants, so that neither knows which treatment group participants are in. Randomisation and blinding are very important in this type of trial to reduce the risk of false results.

For more information on clinical trials and why they are needed, see "Testing Treatments".
http://www.jameslindlibrary.org/pdf/testing-treatments.pdf

What is a placebo?

The placebo group has sugar pills only. The placebo group is used to indicate the effects of having no antibiotic treatment. Any differences in the groups who receive one or both antibiotics are therefore likely to be due to the antibiotic(s). Researchers can then compare the effects of having one of the antibiotics, both antibiotics together, or having nothing.

Why is randomisation needed?

Comparisons of treatments are unfair if the patients in different treatment groups are not similar. The treatment groups must be composed of people who are similar to each other across all the groups - it's no good if one treatment group is mostly full of, say, older people and another is mostly full of younger people, because then any comparison of the different treatments will not be fair. If doctors and patients choose treatments themselves, they can introduce biases – for example, older, sicker patients might choose one treatment and younger, healthier patients might choose another, leading to unfair comparisons. This can lead to dangerous and misleading results.

For more information on randomisation and why it is needed, see "Testing Treatments".
http://www.jameslindlibrary.org/pdf/testing-treatments.pdf

Who can I contact?

If you want to discuss these results further,

- contact your GP (who has been sent a letter about these findings)
- or the Helpline on 0800 085 2411. The helpline number will be available in the short term between 9.30am and 4.30pm.

Where can I find the scientific papers?

The papers are available from the ORACLE Children Study website (http://www.le.ac.uk/oraclechildren). The papers are:

- Kenyon S, Pike K, Jones DR, Brocklehurst P, Marlow N, Salt A, Taylor DJ. Childhood outcomes following the prescription of antibiotics to pregnant women with preterm rupture of the membranes: 7 years follow-up of the ORACLE I trial.
- Kenyon S, Pike K, Jones DR, Brocklehurst P, Marlow N, Salt A, Taylor DJ. Childhood outcomes following the prescription of antibiotics to pregnant women with spontaneous preterm labour: 7 years follow-up of the ORACLE II trial.

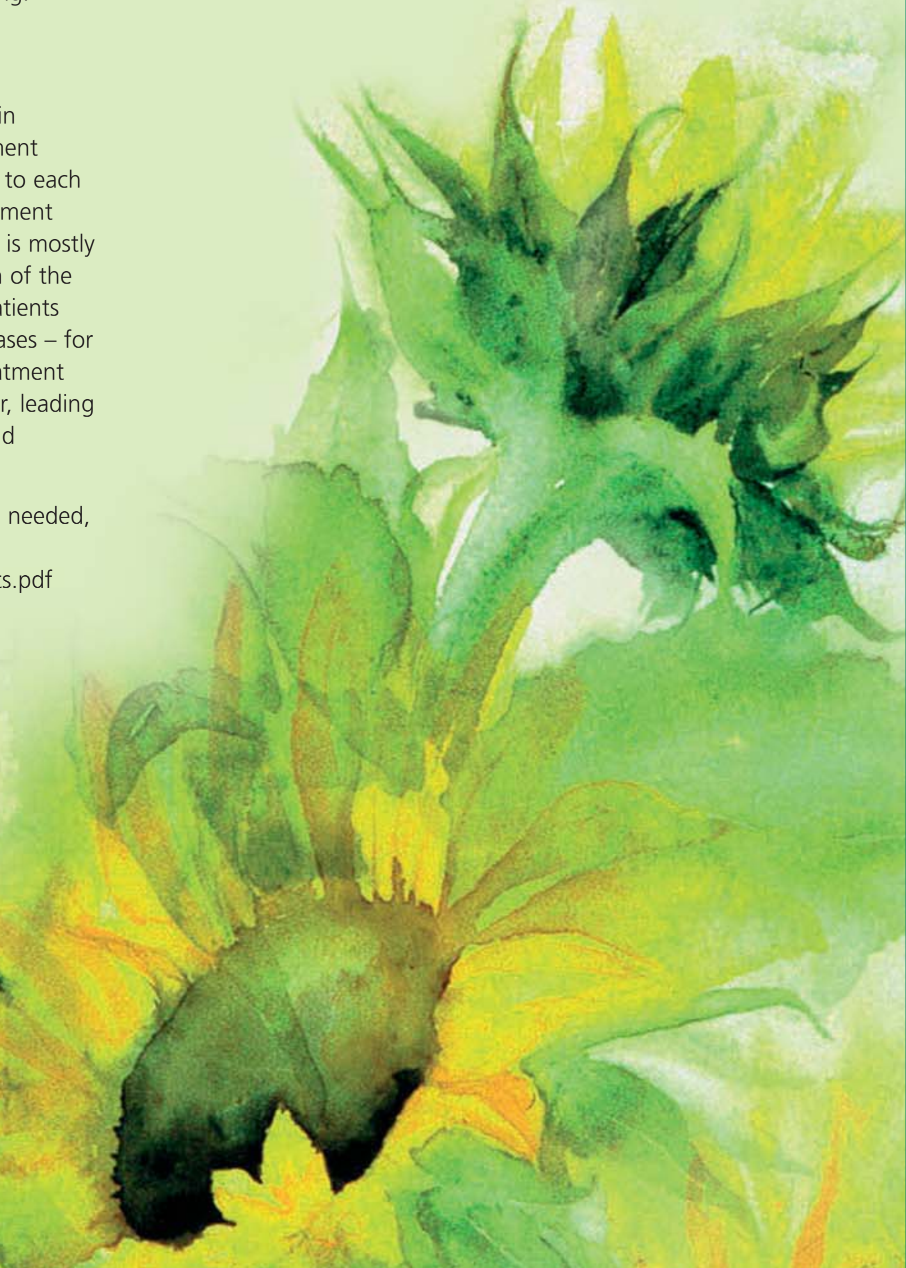

The ORACLE Children Study
22-28 Princess Road West
Leicester LE1 6TP
Telephone: 0116 252 5476
Fax: 0116 252 3277
Email: oracle@le.ac.uk
http://www.le.ac.uk/oraclechildren
